# The Role of Collaboration Between Electrophysiologists and Surgeons in the Management of Complex Arrhythmia Patients

**DOI:** 10.19102/icrm.2019.100203

**Published:** 2019-02-15

**Authors:** Carola Gianni, Chintan Trivedi, Sanghamitra Mohanty, Andrea Natale

**Affiliations:** ^1^Texas Cardiac Arrhythmia Institute, St. David’s Medical Center, Austin, TX, USA; ^2^UOC Cardiologia, IRCCS Ospedale Maggiore Policlinico, University of Milan, Milan, Italy; ^3^Dell Medical School, University of Texas, Austin, TX, USA; ^4^Interventional Electrophysiology, Scripps Clinic, La Jolla, CA, USA; ^5^MetroHealth Medical Center, Case Western Reserve University School of Medicine, Cleveland, OH, USA; ^6^Division of Cardiology, Stanford University, Stanford, CA, USA; ^7^Electrophysiology and Arrhythmia Services, California Pacific Medical Center, San Francisco, CA, USA

**Keywords:** Atrial fibrillation, cardiac implantable electronic device extraction, ventricular tachycardia

## Abstract

Although the need for surgery in patients with arrhythmias has declined in the past several decades due to the emergence of catheter ablation, there is still room for collaboration between electrophysiologists and surgeons, mainly when managing patients with atrial fibrillation, ventricular tachycardia, and cardiac implantable electronic devices.

## Introduction

Interventional electrophysiology was born in the 1970s in the operating room, representing a time when cardiothoracic surgeons and electrophysiologists (EPs) worked closely together to develop a technique for resecting accessory pathways and curing Wolff–Parkinson–White syndrome.^1^ In the following years, cardiothoracic surgeons pioneered the field of interventional electrophysiology by developing effective strategies for the treatment of other forms of supraventricular and ventricular arrhythmias.^2^ Indeed, catheter ablation grew and thrived based on the success observed in patients undergoing antiarrhythmic surgery. Although the need for surgery in patients with arrhythmias has declined in the past several decades, there is still room for collaboration between EPs and surgeons, mainly in managing patients with atrial fibrillation (AF), ventricular tachycardia (VT), and cardiac implantable electronic devices (CIEDs) **(Table 1)**.

## Atrial fibrillation

Surgery for AF comprises both rhythm control and stroke prevention. Currently, the performance of ablation for rhythm control of AF is recommended only in patients undergoing concomitant heart surgery.^3^ “Surgical AF ablation” is a generic term, and there are many associated techniques that have been developed over the years. Although the first iteration of surgical AF ablation—the cut-and-sew, biatrial Cox maze procedure—was highly successful, its technical complexity and prolonged necessary time on cardiopulmonary bypass led to the subsequent introduction of simpler surgical techniques.^4^ These span from minithoracotomy or thoracoscopic, off-pump, left atrial (LA) epicardial procedures to open-chest, on-pump, endo–epicardial biatrial approaches. In all, the cut-and-sew technique has been exchanged for the use of ablation-based tools to create linear lesions that replace the original incisions. These ablation-based tools use many different energy sources, with cryoenergy and bipolar radiofrequency (RF) energy being the most commonly employed and with both being capable of creating consistent transmural lesions.^5^ The lesion set varies in accordance with the surgeon, patient, and surgical approach, ranging from simple pulmonary vein (PV) isolation either alone or in association with other LA lesions (such as posterior wall box isolation and mitral isthmus line) to the more comprehensive Cox maze IV procedure, in which LA lesions are performed along with right atrial lesions and LA appendage (LAA) excision.^6^

An important aspect of surgical ablation to consider is that the most effective ablation set (endo–epicardial Cox maze IV) can only be obtained with the most invasive approach (on-pump, open-chest), thus limiting its application as a standalone procedure for AF. In addition, AF surgery is mainly an empirical procedure, which aims to isolate the PVs and reduce the chance of AF perpetuation by compartmentalizing the atria and for which there are two main considerations to take into account. First, regardless of the lesion set or energy source, there are no intraoperative tools to confirm electrical isolation or conduction block across a line, and gaps represent a major factor of atrial arrhythmia recurrence following surgery.^7^ Second, there are also no tools for intraoperative mapping of AF triggers, thus precluding a tailored ablation targeting patient-specific determinants of AF. Moreover, some areas that show a high prevalence of non-PV triggers (eg, coronary sinus, LAA) and that can be targeted with empirical ablation are not effectively addressed during surgery: even when performing a Cox maze IV procedure, epicardial cryoablation of the proximal coronary sinus does not result in its full isolation and, if there is a residual LAA (“stump”), this is a known arrhythmogenic site.^7^ To overcome this, some advocate for a hybrid approach, in which percutaneous endocardial mapping and ablation is associated with (typically thoracoscopic or subxiphoid pericardioscopic) epicardial surgical ablation. However, in our experience, this increases complication rates without improving outcomes as compared with following extensive endocardial ablation performed by experienced operators using high-power, short-duration RF energy.^8^ To conclude, these are important limitations that can hinder outcomes in nonparoxysmal AF patients, which is the typical AF population undergoing concomitant cardiac surgery for valve or ischemic heart disease. Therefore, although it might be reasonable to perform AF surgery in this cohort, it is important to know about these limitations and to implement adequate postoperative rhythm monitoring to detect recurrences, which can be effectively addressed by catheter ablation.^7^

For stroke prevention, LAA closure is the standard of care in patients with AF undergoing concomitant heart surgery.^3^ There are many approaches and techniques for LAA closure, which can be divided into excision (total removal of the LAA) and exclusion (closure of the ostium of the LAA, which is left in place). As with ablation, the most effective method to achieve LAA closure is using the most invasive approach: to eliminate the risk of an incomplete closure (which can result in “leaks” or a residual stump), LAA excision is performed, followed by endocardial suturing. Endocardial LAA suture/stapling exclusion should avoided, as it carries the highest risk of leaks.^9^ When performing surgical epicardial-only procedures, LAA closure can be obtained by suture ligation, stapled excision, or clipping; however, these all carry a nonnegligible risk of leaks (following ligation) or residual stump (following excision or clipping), potentially limiting the effectiveness of surgical LAA exclusion. It is important to note that, to date, no study of surgical LAA closure has shown a clear benefit with regard to stroke prevention, given the non-negligible incidence of incomplete closure observed in this population.^9^ Therefore, when planning for surgical LAA closure, choosing the proper technique and setup is important in order to adequately follow up with every patient with transesophageal echocardiography.

## Ventricular tachycardia

Encircling endocardial ventriculotomy and subendocardial resection have been performed for decades as a surgical treatment of refractory, scar-related VT.^2^ After the advent of catheter ablation and implantable cardioverter-defibrillators (ICDs), which represent lower-risk alternative therapies, there was a shift away from the surgical treatment of VT. However, a surgical approach to scar-related VT might be the only feasible alternative to catheter ablation in the case of epicardial VT and difficult pericardial access (eg, due to adhesions from prior cardiac surgery or extensive epicardial ablation) or multiple prior ablations that failed as a result of the presence of deep intramural substrate. It may also constitute a reasonable addition for those patients with recurrent refractory VT undergoing open-heart surgery for other cardiac conditions.^10^

In the case of epicardial-only surgical ablation procedures, access can be minimally invasive, achieved either by way of a subxiphoid window or with limited anterior or left thoracotomy, enabling preferential exposure of the inferior versus anterior/lateral left ventricular (LV) walls. This minimally invasive approach is also used for hybrid VT ablation procedures, in which access is obtained by the cardiac surgeon, and the ablation (activation- and substrate-based) is subsequently performed by the EP using an electroanatomical mapping system and an RF ablation catheter, as is usual.^11^ Alternatively, full median sternotomy may be used to expose the whole heart and—if necessary—the endocardial surface. Pure surgical ablation is mainly a substrate-based ablation procedure, due to the difficulty of inducing the clinical arrhythmia in this setting and the impossibility of comparing the electrocardiogram morphology, given the shift of the heart during open-heart surgery. Visual inspection can be used to detect the scar. In endocardial procedures, the scar is usually easily visible and can be targeted with resection, encircling ventriculotomy, or ablation (usually with cryoenergy, which is capable of obtaining large lesions in the cold cardioplegic heart). Meanwhile, epicardially, scar visualization might be hindered by the presence of fat, and EPs might assist by performing intraprocedural electroanatomic mapping to locate areas of low voltage that can then be targeted by cryoablation.

Finally, surgery for VT might aim at neuromodulation with left or bilateral stellate ganglionectomy, which is performed by thoracic surgeons and has been found to be beneficial in selected patients with refractory ventricular arrhythmias and long QT syndrome, idiopathic ventricular fibrillation, or catecholaminergic polymorphic VT.^12^

## Cardiac implantable electronic devices

Although the era of thoracotomy/epicardial lead systems is long gone, cardiothoracic surgeons still play an important role in the management of patients with CIEDs.

Surgical epicardial placement of LV leads for cardiac resynchronization therapy (CRT) is a viable alternative for patients in whom placement via the coronary sinus has failed or in those who remain nonresponders despite multisite or LV endocardial pacing. This can be obtained with limited left thoracotomy, thoracoscopy, or via a subxiphoid approach. Although the former provides better exposure of the lateral wall of the LV, the latter two are less invasive with limited morbidity and should be given preferential consideration. One of the advantages of surgical LV lead implantation is the ability to position the lead anywhere over the lateral wall (scar and/or fat permitting) without the associated anatomical constraints posed by the coronary venous system.^13^ Therefore, when surgically implanting an epicardial lead, instead of blindly positioning the lead in a posterolateral location, it is important to perform mapping to detect the latest area of LV activation, as assessed by the longest sensed right ventricle to LV interval.

The most important and direct way EPs and surgeons collaborate with one another in CIED procedures is during lead extraction. Although the advent of laser technologies has reduced the need for open-chest surgical lead extraction (with a few exceptions, as described below), surgeons are vital to the successful management of complications related to this procedure. With the exception of low-risk cases (ie, a lead less than one year old), lead extraction should be performed in a hybrid room, with a cardiac surgeon available on standby (ie, in the same room) and the necessary equipment to perform an emergent sternotomy/thoracotomy and extracorporeal circulation also available in the room.^14^ Indeed, the most dreaded complication of lead extraction, superior vena cava (SVC) laceration, can be fatal within a few minutes if the chest is not opened to control the bleeding. As a bridging measure, a dedicated 8-cm balloon can be used for endovascular tamponade of SVC bleeding: this allows for the limiting of blood loss and sustaining of hemodynamics, until definite open-chest surgical repair can be performed.^15^ Similarly, cardiac avulsion and tear in the context of lead extraction might require surgical repair, although pericardiocentesis and continuous drainage (with or without self-transfusion) are effective in stabilizing the patient before surgery and might be the only required intervention in the case of small tears. Finally, first-line surgical extraction is still indicated in patients with epicardial leads, extravascular leads (those going through the venous or myocardial wall), and those with large vegetations (ie, those measuring more than 1–2 cm). To curtail morbidity, limited thoracotomy with a transatrial approach can be used: in this situation, lead(s) are grasped via the atriotomy and removed with direct traction or—in the case of dense fibrous encapsulating tissue around the lead—with the aid of a locking stylet and sheath while employing countertraction techniques.^16^

## Limitations

There are few studies in existence that have reported the outcomes of these collaborative procedures, the efficacy/safety of which are highly dependent on the relative skills of the EP and the surgeon and also their ability to work together. Moreover, this collaboration is most fruitful when dealing with complex patients, where less invasive approaches that typically carry lower morbidity have already failed. Therefore, it is difficult to provide definite recommendations; nonetheless, herein, we aimed to give an overview of how EPs and cardiac surgeons can collaborate, describing options that have been effectively and safely employed when dealing with complex arrhythmic patients.

## Conclusions

Close collaboration between EPs and cardiac surgeons is important in the management of patients with a wide array of cardiac rhythm disorders. Recognizing the limitations and advantages of the respective existing approaches is key to ensure a fruitful collaboration.

## Figures and Tables

**Table 1:** Collaboration between EPs and Cardiac Surgeons

	Approach	Strategy	Comments	
**AF**				
Rhythm control	Surgical only: off-pump, mini-invasive, epicardial-only ablation → on-pump, open-chest, endo–epicardial ablation	PVI only → + LA lines → + RA lines → full Cox maze IV (including LAA exclusion)	•	Reasonable addition in patients with AF undergoing concomitant cardiac surgery
			•	Limited role as a standalone procedure
			•	Empirical only ○ No intraoperative confirmation of effective lesion formation ○ Cannot target patient-specific triggers
	Hybrid: transcatheter endocardial ablation + off-pump, mini-invasive epicardial ablation	PVI + LA and RA lines	•	Minimally invasive
			•	Might improve outcomes in nonparoxysmal AF versus PVI only
			•	Not better nor safer than AF ablation performed by experienced EPs
Stroke reduction	Off-pump, mini-invasive, epicardial-only access → on-pump, open-chest, endo–epicardial access	LAA stapled excision, LAA clipping → LAA exclusion with endocardial stapling/suture), LAA excision with endocardial suturing	•	Can be minimally invasive or performed during concomitant cardiac surgery
			•	Relative high prevalence of incomplete occlusion ○ Leaks can lead to higher thromboembolic risk and it is important to establish follow-up surveillance with TEE ○ Residual stumps following exclusion are still arrhythmogenic
**VT**				
	Surgical only: off-pump, mini-invasive, epicardial-only ablation → on-pump, open-chest, endo–epicardial ablation	Epicardial-only substrate-based ablation (guided by EAM) → + endocardial excision (guided by visual inspection)	•	Second-line treatment after failed epicardial access/ablation
			•	Reasonable addition in patients undergoing concomitant cardiac surgery
			•	Empirical only ○ Difficult to induce arrhythmias during general anesthesia ○ Hard to compare ECG morphology with the clinical VT (due to heart shift)
	Hybrid: limited thoracotomy or subxiphoid window epicardial access + transcatheter ablation	Activation- and substrate-based ablation guided by EAM	•	Minimally invasive
			•	Allows for EAM in cases with difficult pericardial access (adhesions)
			•	Difficult to induce arrhythmias during general anesthesia
	Neuromodulation: thoracotomy (thoracic surgeon)	Left or bilateral stellate ganglionectomy	•	Refractory ventricular arrhythmias and long QT syndrome, idiopathic ventricular fibrillation, or catecholaminergic polymorphic VT
			•	Not indicated as first-line treatment
**CIEDs**				
CRT	Thoracoscopy, limited thoracotomy, or subxiphoid window	LV lead positioning in the area of longest sensed RV–LV interval	•	Minimally invasive
			•	Alternative after failed placement via the coronary sinus or in nonresponders despite multisite or LV endocardial pacing
			•	Allows for lead positioning anywhere in the epicardial LV without the constraints of the coronary venous system
Lead extraction	Open-chest	Surgical repair of SVC laceration or large myocardial tear	•	High-risk (lead placement for more than one year) lead extractions should be performed in hybrid rooms, with the cardiac surgeon and equipment to perform emergent sternotomy/thoracotomy/extracorporeal circulation available and ready in the same room
	Limited thoracotomy → + atriotomy	Epicardial lead extraction → transatrial lead extraction	•	First-line treatment for epicardial leads, extravascular leads, and those with large (; 1–2 cm) vegetations

AF: atrial fibrillation; CIED: cardiac implantable electronic device; CRT: cardiac resynchronization therapy; EAM: electoanatomic mapping; ECG: electrocardiogram; EP: electrophysiologist; LA: left atrium/atrial; LAA: left atrial appendage; LV: left ventricle/ventricular; PVI: pulmonary vein isolation; RA: right atrium/atrial; SVC: superior vena cava; TEE: transesophageal echocardiography; VT: ventricular tachycardia.
